# Early Adoption of Thyroid Radiofrequency Ablation in Canada: Physician Experiences, Barriers, and Facilitators to Implementation

**DOI:** 10.1177/19160216261451820

**Published:** 2026-06-18

**Authors:** Harrison Gao, David Liu, Ben B. Levy, Justin Shapiro, Kevin M. Higgins, Diana E. Khalil, Elizabeth E. Cottrill, Courtney Poon, Pabiththa Kamalraj, Justine Philteos, Antoine Eskander

**Affiliations:** 1 Temerty Faculty of Medicine, University of Toronto, Toronto, ON, Canada; 2 Institute of Health Policy, Management, and Evaluation, University of Toronto, Toronto, ON, Canada; 3 Sunnybrook Research Institute, Sunnybrook Health Sciences Centre, Toronto, ON, Canada; 4 Department of Medicine, Queen’s University, Kingston, ON, Canada; 5 Department of Otolaryngology – Head & Neck Surgery, University of Toronto, Toronto, ON, Canada; 6 Department of Otolaryngology – Head & Neck Surgery, Western University, London, ON, Canada; 7 Department of Otolaryngology – Head & Neck Surgery, Sunnybrook Health Sciences Centre, Toronto, ON, Canada; 8 Department of Otolaryngology – Head & Neck Surgery, Waterloo Regional Health Network, Waterloo, ON, Canada; 9 Department of Otolaryngology – Head & Neck Surgery, Thomas Jefferson University, Philadelphia, PA, USA; 10 Department of Otolaryngology – Head & Neck Surgery, Harvard Medical School, Boston, MA, USA; 11 Department of Otolaryngology – Head & Neck Surgery, Michael Garron Hospital, Toronto, ON, Canada

**Keywords:** thyroid radiofrequency ablation, thyroid nodule, health policy, physician experiences, barriers and facilitators

## Abstract

**Importance:**

Thyroid radiofrequency ablation (RFA) is a minimally invasive alternative to surgery for thyroid nodules. Despite strong international evidence, thyroid RFA was only approved by Health Canada in April 2023. Understanding the experiences of early adopters can inform the broader adoption of RFA in Canada and other healthcare systems.

**Objective:**

To describe the implementation experiences of early adopters of thyroid RFA in Canada and identify barriers and facilitators to adoption.

**Design:**

Multiple methods.

**Setting:**

All 8 listed RFA facilities in Canada.

**Participants:**

Physicians performing thyroid RFA (n = 9).

**Main Outcome Measures:**

Survey and semi-structured interviews.

**Results:**

Most participants reported low RFA volumes (median [IQR] = 2.0 [0.6-3.0] cases/month) and short wait times (1.5 [1.0-2.0] months). For thyroid surgery on similar nodules, participants performed higher volumes (7.5 [6.0-10.2] cases/month, *P* = .01) and had longer wait times (6.0 [5.5-8.5] months, *P* < .01). Four major themes emerged: (1) financial barriers and inequity; (2) impact on the healthcare system; (3) learning curve; and (4) motivators and facilitators. Financial barriers were driven by the RFA generator ($40 500 CAD) and single-use probes ($1500-$2500 CAD). RFA was funded privately out-of-pocket in most physician practices (n = 7) and rarely publicly covered (n = 2). Motivators and facilitators included patient-centred benefits, professional development, institutional support, and potential resource savings.

**Conclusions:**

Thyroid RFA adoption in Canada is in its infancy, characterized by low procedural volumes, few providers, and geographical disparities. Early adopters reported positive experiences with RFA. However, inconsistent funding models, billing codes, and policy frameworks have resulted in inequitable access for patients.

**Relevance:**

Many healthcare systems are in the early stages of RFA adoption, similar to Canada. This study identifies key experiences, barriers, and facilitators applicable to physicians, leaders, and policymakers globally who are interested in adopting RFA.

## Graphical abstract

**Graphical Abstract:**
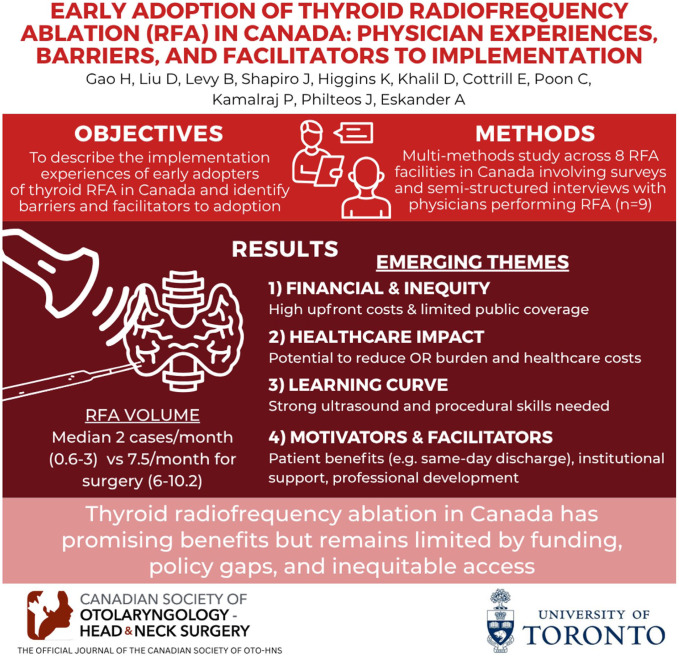

## Key Messages

1. Thyroid radiofrequency ablation (RFA) adoption in Canada is still in its infancy, characterized by low procedural volumes, a small number of providers, and significant geographical access gaps.
2. Early adopter experiences with thyroid RFA demonstrate substantial patient-centred benefits, professional enthusiasm, and potential to alleviate the burden on the healthcare system.
3. Key policy and practice gaps include expanding access and public coverage, defining clear evidence-based criteria for patient selection, and implementing appropriately valued billing codes.

## Introduction

Thyroid nodules are detectable on ultrasonography in 19% to 67% of individuals.^1-4^ Benign nodules account for nearly 90% of all thyroid nodules and are typically monitored without intervention if they remain asymptomatic.^1,4^ However, some benign nodules cause compressive symptoms (neck discomfort, globus sensation, dysphagia, dyspnoea) or cosmetic concerns due to their size. Most symptomatic benign nodules are treated with surgery, which carries the risk of hypothyroidism, recurrent laryngeal nerve injury, and scarring.^5,6^

Thyroid radiofrequency ablation (RFA) is a minimally invasive outpatient treatment for thyroid nodules with significant advantages over traditional surgical resection.^7,8^ RFA treatment may yield up to 70% to 90% nodule volume reduction at 12 months, improved local symptoms, and cosmetic improvements.^5^ It also avoids the risks of general anaesthesia and scarring with a much lower risk of hypothyroidism and nerve injury.^7,9,10^ RFA is a widely accepted treatment for benign symptomatic thyroid nodules and hyperfunctioning nodules.^6,9,11^ For small papillary thyroid cancers, RFA is increasingly considered according to recent 2025 Korean Society of Thyroid Radiology guidelines and 2025 American Thyroid Association guidelines.^12,13^

Despite strong international adoption since it was first performed in 2002, thyroid RFA only received Health Canada approval in April 2023, highlighting a substantial delay in its adoption and dissemination.^14^ The Canadian healthcare system may uniquely benefit from thyroid RFA as long wait times reveal poor access to timely elective surgical care due to limited operating room availability.^15,16^

The implementation of RFA by interventional thyroidologists (including otolaryngologists, general surgeons, radiologists, and endocrinologists) requires navigating a complex landscape of barriers and facilitators. Globally, many healthcare systems are in the early stages of RFA adoption, similar to Canada. As interest in RFA grows and more physicians consider its use, the experiences of early adopters are essential for guiding the broader adoption of RFA in the Canadian system and other healthcare systems that may follow suit. Therefore, the objectives of this study were (1) to describe the real-world implementation experiences of early adopters of thyroid RFA in Canada; and (2) to identify barriers and facilitators to the adoption of thyroid RFA.

## Methods

This project was approved by the Sunnybrook Health Sciences Centre Research Ethics Board (REB #6587). We conducted a multiple-methods study comprising a structured survey and semi-structured interviews.^17^ A qualitative descriptive approach was used to generate practical, clinically relevant insights from participant experiences without extensive inference or theoretical interpretation.^18,19^ The Consolidated Criteria for Reporting Qualitative Research (COREQ) 32-item checklist was used as a framework for study design, execution, and reporting.^20^ The COREQ checklist is available under Supplemental Material 1.

### Participant Recruitment

A purposive sampling strategy was used to identify early adopters of thyroid RFA in Canada. Any physician performing thyroid RFA in Canada was eligible to participate. Our sampling strategy ensured diverse representation across geographical regions, physician specialties, and experience levels with RFA. At least one participant was recruited from each of the 8 listed thyroid RFA facilities in Canada at the time of this study. The 8 facilities were identified through the Thyroid RFA Facilities of Canada webpage and the North American Society for Interventional Thyroidology online directory.^21,22^ One additional early adopter, who was not listed, was identified through the professional network of the principal investigator (A.E.).

Participants were contacted by email by the lead author (H.G.) and written consent was obtained. Nine out of 12 physicians contacted agreed to participate and the remaining 3 did not respond. The survey and interview guide were pilot tested with 3 otolaryngologist–head and neck surgeons (2 RFA users and 1 nonuser) who did not participate in the study.

### Data Collection

Data were collected using a 25-item online survey (REDCap) and a 30-minute semi-structured Zoom interview from March to August 2025.^23,24^ The survey and interview guide are available in Supplemental Material 2. All interviews were conducted by one author (H.G.), audio recorded using Zoom, and transcribed using third-party transcription software. No prior relationships existed between the interviewer and the participants. Participants were offered the opportunity to review and revise their transcripts. When no new themes emerged during iterative thematic analysis, data saturation was reached and study recruitment concluded.^25^

### Data Analysis

**Figure 1.**
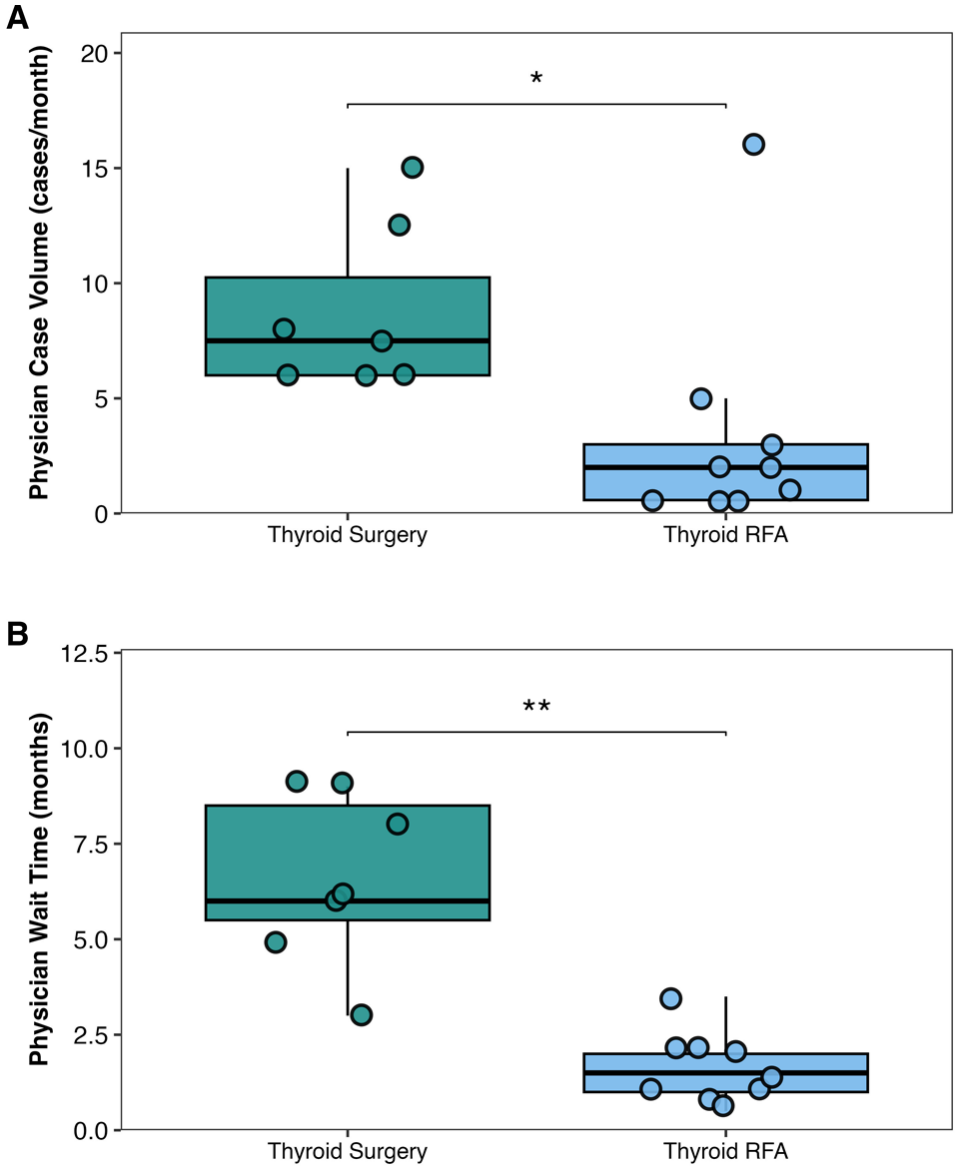
(A) Case volumes and (B) wait times for thyroid surgery compared to thyroid RFA. Thyroid surgery wait times were reported for symptomatic benign or hyperfunctioning nodules. Each box plot depicts the median, interquartile range, and individual data points. RFA, radiofrequency ablation. *P < .05, **P < .01.

Survey responses were anonymized and descriptive statistics were generated using RStudio, R version 4.3.0.^26^ Demographics were reported in aggregate to protect confidentiality, as there are very few thyroid RFA users in Canada. For instance, 2 provinces only had a single RFA user; therefore, these were grouped as “other provinces” (Table 1). Mann–Whitney *U* tests were used to compare case volumes and wait times across thyroid surgery and RFA (Figure 1).

**Table 1.** Participant Demographics (n = 9).

Participant characteristic	n (%)
Province	
Ontario	5 (56)
Quebec	2 (22)
Other provinces^a^	2 (22)
Years of independent practice	
<5 years	1 (11)
5-9 years	3 (33)
10-19 years	3 (33)
20-30 years	2 (22)
Primary practice setting	
Academic hospital	6 (67)
Community hospital	3 (33)
Specialty	
Otolaryngology – head and neck surgery	4 (44)
General surgery	3 (33)
Radiology	1 (11)
Endocrinology	1 (11)
RFA procedural setting	
Minor procedure room (hospital)	6 (67)
Office/clinic	2 (22)
Operating room (private surgery centre, procedure done under local anaesthesia)	1 (11)

Abbreviation: RFA, radiofrequency ablation.

a Other provinces represents 2 distinct provinces, each with 1 single RFA provider. Their data are reported in aggregate to protect participant privacy.

**Figure 2.**
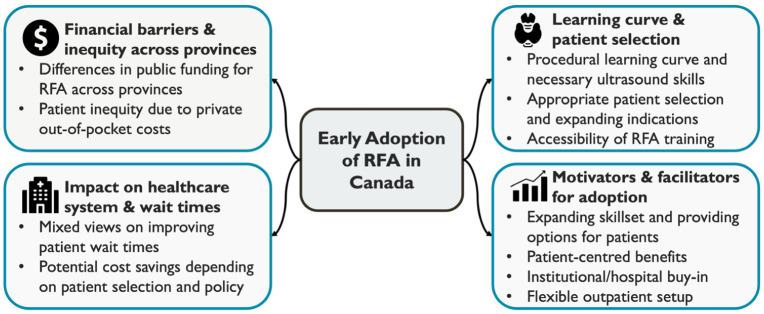
Main themes and subthemes from qualitative interviews on the early adoption of thyroid RFA in Canada. RFA, radiofrequency ablation.

Interview transcripts were analyzed thematically through an inductive–deductive approach.^27^ A preliminary coding framework was first developed through a literature review and discussions with the senior author and content expert (A.E.); then, 2 coders (H.G. and D.L.) independently reviewed and coded all interview transcripts in duplicate using NVivo 14. Consensus discussions between the coders and the senior author were used to generate themes and subthemes. For reflexivity and positionality, H.G. and D.L. were medical students and graduate students at 2 Canadian universities at the time of the interviews. A.E. is a head and neck surgical oncologist, health services researcher, and early adopter of thyroid RFA in Canada (Figure 2).

## Results

Nine participants were included in this study. All 8 listed RFA facilities in Canada, as well as one newly established centre that had not yet been listed, were represented in our sample. Participants practiced across 9 different sites, 4 provinces, and 4 specialties (Table 1). Most participants performed RFA in minor procedure rooms (n = 6 [67%]), while others utilized clinic settings or private operating rooms under local anaesthesia (Table 1).

Most participants reported low RFA volumes (median [IQR] = 2.0 [0.6-3.0] cases/month) with short wait times (median [IQR] = 1.5 [1.0-2.0] months). For thyroid surgery on similar nodules, participants reported higher volumes (median [IQR] = 7.5 [6.0-10.2] cases/month, *P* = .01) with longer wait times (median [IQR] = 6.0 [5.5-8.5] months, *P* < .01) compared to RFA (Figure 1).

### Theme 1: Financial Barriers and Inequity Across Provinces

Financial barriers were the most significant challenge faced during RFA adoption. Participants described 3 main cost components of RFA: the RFA generator ($40 500 CAD), single-use disposable probes ($1500-$2500 CAD), and the physician remuneration fee (variable). There was only one medical device supplier for RFA in Canada. Additional costs included local anaesthetic, syringes, draping, and other supplies. RFA was funded privately out-of-pocket in most physician practices (n = 7) and rarely publicly covered (n = 2) (Table 2).

**Table 2.** Funding Disparities Across Provinces, Showing Differences in Coverage for RFA Generator, Single-Use Probe Costs, and Physician Remuneration Based on Interviews.

Province (number of participants)	RFA generator cost	Single-use probe cost	Physician remuneration
Ontario (n = 5)	Private	Private	Mixed opinions on private vs public^a^
Quebec (n = 2)	Private	Private	Private
Other provinces (n = 2)	Public or philanthropic donations	Public	Public

Abbreviation: RFA, radiofrequency ablation.

Private funding refers to out-of-pocket expenses for patients. Public refers to programs funded by the Ministry of Health or hospital budgets. “Other provinces” represents 2 separate provinces, each with 1 single RFA provider.

a Some participants in Ontario use the public billing code (J069) while others do not.

In Ontario and Quebec, where RFA was privately funded, most participants used a rent-to-own model for the generator rather than purchasing it outright. This added a $1000 CAD machine rental surcharge to each probe until a certain number of cases was reached. Then, participants would receive a new machine at no additional cost. P9 explained why they chose the rent-to-own model:

I don’t have that startup cost. . . until I see enough volume for it, I just can’t justify it. . . [medical device company has] a fairly good model about renting the machine, it’s called rent-to-own.

In publicly funded settings, some participants received philanthropic donations or hospital foundation funding for the generator. For participants with public funding, funding was procured on a centre-by-centre basis, and there were no provincial mandates in place. Coverage depended on whether a specific facility or hospital was willing to fund the probes. P7 reported:

The stars aligned that there was a new outpatient centre. . . they were able to advocate to include thyroid RFA in their budget.

Many participants identified the disposable probe as the most significant cost barrier to the adoption of RFA. P4, who worked in a private RFA practice, noted:

The issue is the probe. . . it’s not in the budget of the hospital. . . unless there was some mandate from [provincial ministry of health] to say, ‘hospitals pay for this probe. . . and we’ll supplement your budget to do that’.

The final component was physician remuneration, which represented a relatively small portion of the total cost. Most patients were charged privately when no alternative funding existed. In Ontario, a billing code is available for percutaneous focal thermal ablation of solid tumours (J069) to reimburse RFA for liver cancer.^28^ However, there was considerable debate on whether this code could apply to thyroid RFA (Table 2). Across all provinces, participants reported a lack of guidelines on remuneration, leaving policy open to interpretation. P4 described using the. . . “*[provincial health insurance plan] generated fee code [J069], which I believe exists currently. . . I think there needs to be some clarification. . .*”

When discussing the creation of billing codes, participants were concerned about whether policymakers would assign appropriate values. P4 cautioned that the recently created American relative value unit (RVU) code was “*based on fine needle aspiration. . . paid quite low*”. P3 shared a similar sentiment:

Unless they make [billing code] similar to. . . a thyroid surgery, it’s hard to justify because the time required [for RFA] is about the same as a hemithyroidectomy.

Ultimately, the financial barriers highlighted patient inequity driven by the lack of public funding and formal policy supporting RFA, particularly in Ontario and Quebec (Table 2). Early adopters uniformly expressed a desire for public coverage and concern about the lack of equitable access. As P6 stated:

My patients at [hospital] can’t afford the thousands of dollars for this. . . Only patients who can afford this treatment can benefit from this minimally invasive approach, which doesn’t fit well with our Canadian healthcare model.

### Theme 2: Impact on Healthcare System and Wait Times

Participants expressed mixed views on whether RFA reduces strain on the healthcare system and shortens wait times. Some saw it positively as an alternative to reduce costs within the surgical system. P2 noted:

I think it opens another avenue to take the burden off the system in terms of saving. . . operating room time, saving costs. . . for all the support staff and anesthesiologist and potentially a hospital bed stay.

Others felt that there would be little to no impact on surgical waitlists and cautioned that some patients who were never interested in surgery might seek RFA instead, adding costs without reducing surgical demand. P5 explained:

I don’t think wait time for surgery is impacted by RFA. . . they’re two completely different subsets of patients. The patients who want surgery. . . aren’t interested in RFA, and those who want RFA. . . specifically seek it out because they don’t want an operation.

Overall, participants agreed that RFA had the potential to save healthcare resources. However, whether this becomes reality would depend on how physicians adopt it into practice.

### Theme 3: Learning Curve and Patient Selection

Early adopters stressed that RFA required advanced ultrasound skills and described a learning curve for both the procedure and patient selection. P9 noted:

You have to be a really good ultrasonographer for you to be able to do this technique well. And there’s a big learning curve. For me, I was doing ultrasounds for years, biopsies for years. I have thousands. . . under my belt. . . for [people who don’t do much thyroid surgery or ultrasound] to dabble in RFA, there’s going to be some concerns.

Participants emphasized the importance of careful patient selection and expanding indications appropriately. There was broad consensus for use on symptomatic benign nodules and hyperfunctioning nodules. P5 stated:

There’s a learning curve to the procedure, but I think there’s as important a learning curve to deciding, choosing your patient. . . that’s an education unto itself.

Most participants accessed RFA training in the United States or Korea. A few trained with other early adopters in Canada. P7 shared, “*I went to Toronto to do a day with [another early adopter]. . . I had [also] gone somewhere else just for a short course.*”

### Theme 4: Motivators and Facilitators for Adoption

Participants identified several key motivators and facilitators that encouraged them to adopt RFA. For many, there was a strong interest in expanding their surgical skillset for professional development. P5 explained:

As a surgeon who performs a lot of head and neck endocrine surgery, to not have [RFA] as part of my armamentarium is a little bit of a chink in the armour, or a weapon missing.

Perceived patient-centred benefits were also a strong motivator, with participants emphasizing the advantages of RFA over surgery. There was also discussion on quality of life and satisfaction. As P6 described:

Patients go home the same day, they have no incision, no general anesthetic, decreased chance of hypothyroidism, low risk to the recurrent laryngeal nerve.

One major facilitator was institutional buy-in, which provided access to a portable ultrasound, nursing time, and procedure space. P2, who worked in a publicly covered RFA practice, recalled: “*I just proposed [RFA] to the head of my department. . . I said, ‘this is the way the research is going, and I think it is better for patients. Let’s get this machine and start doing them’. And there was no pushback. It was pretty much a go.*”

Even when participants did not receive institutional support, adoption was still possible in a clinic/office setting. P5 reported, “*if we would have insisted on trying to do it in a hospital setting. . . that would have been a huge barrier.*”

Public funding was a strong facilitator for patient demand. For P2, who worked in a setting where RFA probes are publicly funded, they stated that “*demand has been greater than any other program that I’ve seen started in my department. . . referrals have been coming in steadily.*”

## Discussion

### Current State of RFA Adoption in Canada

Thyroid RFA adoption in Canada is still in its infancy, characterized by low procedural volumes, a small number of providers, and significant geographical access gaps.^22^ When publicly covered, one participant noted that the demand for RFA was greater than any other program they had ever seen in their department. In contrast, in Ontario and Quebec, where RFA was privately funded (out-of-pocket), several participants performed one or fewer RFA cases per month (Figure 1). Two main factors contributed to low demand in the private setting. First, physicians were selective, only performing cases with the highest likelihood of benefit and lowest risk of recurrence. Second, many patients were unable to afford the procedure. Without public funding, the existing private market in Ontario and Quebec appeared small.

Equitable access was a central concern of RFA adopters. Canadian universal healthcare principles are challenged by provincial health systems that function as silos. For the 2 participants working in publicly covered RFA settings, funding for single-use probes, equipment, and nursing support was procured on a centre-by-centre basis as no provincial mandates or guidance existed yet. Each province must independently evaluate, approve, and fund new technologies, often through lengthy health technology assessment processes. The province of Ontario is the first to release its draft health technology assessment of thyroid RFA and to begin developing policy in this area.^29^ As of March 2026, it recommends that RFA be publicly funded; however, this recommendation has not yet been adopted by the Ministry of Health and remains under consideration. Some participants proposed the possibility of hybrid funding models involving private insurance, though this was considered improbable given that most private insurance plans exclude medical procedures. Ultimately, Canadian health policy must reconcile the federal principles of universality with the realities of provincial implementation to enable equitable adoption and access to novel treatment options.

### RFA Setting and Learning Curve

RFA was performed in a variety of clinical settings. An office-based set-up was considered feasible and equivalent to a minor procedure room. The only advantage to a procedure room was that supplies such as local anaesthetic, drapes, and instrument trays may be more easily accessible. One support staff (typically a nurse) was required for the procedure.

All participants agreed that RFA requires ultrasound and procedural expertise. They also emphasized a responsibility to select appropriate patients. Many participants were motivated by the opportunity to expand their skillset and offer patients more options for managing thyroid nodules. The learning curve was a key discussion point as RFA is a technically challenging procedure. According to the American Thyroid Association 2023 statement on ablation for benign nodules, the initial learning phase is typically reached after ~30 cases.^11^ Proficiency is generally achieved after ~60 cases. However, current case volumes suggest that most Canadian practitioners have not yet surpassed the initial learning phase based on volume alone.

Regarding follow-up, participants adhered to guideline-based ultrasound surveillance at 1 to 3, 6, and 12 months during the first year to assess volume reduction and treatment success.^11^ Early adopters expressed interest in expanding their practice to include small papillary cancers or recurrent disease, which are more challenging to treat.

### Barriers and Facilitators

The greatest barrier to RFA adoption was cost. One potential change that would provide hospitals with a clear mandate to adopt RFA is the establishment of appropriate billing codes in each province. Participants also expressed concern that future billing codes should not undervalue RFA, emphasizing that reimbursement should be comparable to that of a hemithyroidectomy, given the similar procedural time and complexity.

Institutional support was another critical component. Most participants performed RFA in a procedure room within a hospital (67%). Others did it in offices/clinics (22%) or private surgery centres (11%) if they were unable to obtain hospital support. Access to procedure space, equipment such as an ultrasound machine, and nursing staff at the hospital were strong facilitators for adoption. Overall, Canadian physicians reported highly positive experiences with RFA despite barriers related to cost, infrastructure, and policy.

### System-Level Implications and Future Directions

The international evidence base for thyroid RFA has expanded rapidly, demonstrating robust safety and efficacy. Large multicentre cohort studies and meta-analyses indicate that RFA may achieve 70% to 90% volume reduction at 12 months and normalization of thyroid function in hyperfunctioning nodules.^5,7,30,31^

Recent evidence also shows that RFA provides cost savings compared to thyroid lobectomy.^32-34^ Theoretically, RFA could be a cost-effective way to alleviate surgical burden, but there was considerable debate among participants. Some felt RFA might attract new patients who are not surgical candidates or originally declined surgical intervention. Participants agreed that the extent to which RFA reduces the burden on the healthcare system ultimately depends on the underlying policy and funding structures governing which patients are eligible.Thyroid RFA is being adopted worldwide, but different countries remain at different phases of its implementation. In the United States, thyroid RFA was approved by the Food and Drug Administration in 2018 and typically costs $5000 to $10 000 USD.^35^ Current Procedural Terminology (CPT) billing codes for RFA were introduced in January 2025. Code 60660 (ablation within a single lobe) is valued at 5.75 work RVUs, while the add-on code 60661 (ablation in an additional lobe) is valued at 4.25 work RVUs.^36^ In the United Kingdom, thyroid RFA was approved by the National Institute for Health and Care Excellence in 2016 and is available in the public and private systems.^37^ Private costs range between 4900 GBP and 8350 GBP and may have shorter wait times.^38^ South Korea remains the global leader, with thousands of procedures performed annually, costing between 1 million to 4 million KRW.^39,40^ Korean public health insurance only publicly covers select cases such as recurrent thyroid cancer.^12^

Compared to other countries, Canada remains behind in adopting thyroid RFA, having only approved the procedure in 2023. Although Canadian Medicare operates under a single-payer system, the lack of consistent public funding places RFA access closer to private payment models in other countries. The low RVU assignment for the newly created CPT codes in the United States highlights the risk of inadequate reimbursement. Many countries offer public coverage for RFA for certain indications, which appears to support broader adoption.

This study has several limitations. The small sample size and focus on early adopters may limit generalizability. Early adopters may have a more favourable perception of RFA compared to later adopters or non-users. Financial incentives may further influence perspectives in settings where RFA is delivered through private, out-of-pocket funding models. Additionally, this study reflects physician perspectives only and does not capture the views of patients, administrators, or policymakers, which could provide additional clinical and health system insights. Finally, data were self-reported and may be subject to recall bias or estimation inaccuracies.

In conclusion, thyroid RFA in Canada is at a pivotal moment in the adoption curve. Early adopter experiences demonstrate strong clinical promise, professional enthusiasm, and potential to alleviate surgical system burden. However, financial, institutional, and policy barriers threaten healthcare equity and sustainable integration of RFA. To move forward, key policy and practice gaps include expanding access, defining clear evidence-based criteria for patient selection, and implementing appropriately valued billing codes. Funding models must also extend beyond physician remuneration to support the full procedural infrastructure, including RFA and ultrasound equipment, disposable costs, and nursing support. With these elements in place, RFA could become a valuable, accessible, and sustainable component of thyroid nodule management within the Canadian healthcare system and other healthcare systems that may follow suit.

## Supplemental Material

sj-docx-1-ohn-10.1177_19160216261451820 – Supplemental material for Early Adoption of Thyroid Radiofrequency Ablation in Canada: Physician Experiences, Barriers, and Facilitators to Implementation Supplemental material, sj-docx-1-ohn-10.1177_19160216261451820 for Early Adoption of Thyroid Radiofrequency Ablation in Canada: Physician Experiences, Barriers, and Facilitators to Implementation by Harrison Gao, David Liu, Ben B. Levy, Justin Shapiro, Kevin M. Higgins, Diana E. Khalil, Elizabeth E. Cottrill, Courtney Poon, Pabiththa Kamalraj, Justine Philteos and Antoine Eskander in Journal of Otolaryngology - Head & Neck Surgery

sj-docx-2-ohn-10.1177_19160216261451820 – Supplemental material for Early Adoption of Thyroid Radiofrequency Ablation in Canada: Physician Experiences, Barriers, and Facilitators to Implementation Supplemental material, sj-docx-2-ohn-10.1177_19160216261451820 for Early Adoption of Thyroid Radiofrequency Ablation in Canada: Physician Experiences, Barriers, and Facilitators to Implementation by Harrison Gao, David Liu, Ben B. Levy, Justin Shapiro, Kevin M. Higgins, Diana E. Khalil, Elizabeth E. Cottrill, Courtney Poon, Pabiththa Kamalraj, Justine Philteos and Antoine Eskander in Journal of Otolaryngology - Head & Neck Surgery
