# Supplementary material for: Early Adoption of Thyroid Radiofrequency Ablation in Canada: Physician Experiences, Barriers, and Facilitators to Implementation
Source: J Otolaryngol Head Neck Surg. 2026 Jun 18;55:19160216261451820. doi: 10.1177/19160216261451820 (PMC13305619; doi:10.1177/19160216261451820)
Supplement: sj-docx-1-ohn-10.1177_19160216261451820 – Supplemental material for Early Adoption of Thyroid Radiofrequency Ablation in Canada: Physician Experiences, Barriers, and Facilitators to Implementation [file sj-docx-1-ohn-10.1177_19160216261451820.docx]

**Survey Questions**

**Demographics:**

1. How many years have you been practicing independently as a licensed medical professional (after completion of residency or fellowship)?
   1. < 5 years
   2. 5-9 years
   3. 10-19 years
   4. 20-30 years
   5. >30 years
2. What province/territory do you practice in?
   1. Alberta
   2. British Columbia
   3. Manitoba
   4. New Brunswick
   5. Newfoundland and Labrador
   6. Northwest Territories
   7. Nova Scotia
   8. Nunavut
   9. Ontario
   10. Prince Edward Island
   11. Quebec
   12. Saskatchewan
   13. Yukon
3. What municipality do you practice in?
   1. ______________(Open Answer)
4. What is the primary setting of your practice?
   1. Academic hospital
   2. Community hospital
   3. Private clinic
   4. Other (please specify):________
5. What is your primary medical/surgical specialty?
   1. Otolaryngology – Head & Neck Surgery
   2. General Surgery
   3. Interventional Radiology
   4. Endocrinology
   5. Other (please specify):________

**Current Thyroid Practice:**

1. Approximately how many thyroid surgical cases do you perform per month?
   1. _______ (open answer)
2. For symptomatic benign nodules or autonomously functioning nodules, what is your average patient wait time for surgical treatment (months)?
   1. _______ (open answer)
3. Approximately how many thyroid radiofrequency ablation (RFA) procedures do you perform per month (# cases)?
4. _______ (open answer)
5. For symptomatic benign nodules or autonomously functioning nodules, what is your average patient wait time for an RFA procedure (months)?
6. _______ (open answer)
7. Where do you conduct your thyroid RFA procedures?
   1. Office/Clinic
   2. Minor Procedure Room (Hospital)
   3. Operating Room
   4. Other (please specify):________

**Semi-Structured Interview Guide**

Current Practice/Perceptions on Thyroid RFA:

1. Please tell me about your current practice as a physician.
   1. Probe: Can you tell me more about your thyroid practice specifically?
   2. Probe: What type of treatments do you offer patients with symptomatic benign thyroid nodules?
   3. Probe: What type of treatments do you offer patients with autonomously functioning thyroid nodules?
   4. Probe: What type of treatments do you offer patients who have cosmetic concerns due to thyroid nodules?
2. What are your perceptions of thyroid RFA as a safe and effective alternative to surgery?
   1. Probe: Do you believe it could improve patient outcomes?
   2. Probe: Do you believe it could improve patient wait times?
3. Overall, what comes to mind when you think about thyroid RFA?
4. Follow-Up Discussion Questions based on Survey Results
   1. If a participant has any notably strong opinions (e.g., strongly agrees or disagrees that thyroid RFA would improve clinical care, has sufficient evidence to support its adoption, etc), then follow-up discussion questions to determine the rationale behind this perception can be asked.
   2. Follow-up discussion question: Could you elaborate on your experiences that inform your perspective on _______ from the survey?
   3. Follow-up discussion question: What factors in your practice contribute to your opinion on _______ from the survey?

Implementation Experiences:

1. Why did you choose to implement thyroid RFA?
2. Please tell me about your experiences implementing thyroid RFA?
   1. Probe: What challenges did you experience with implementing thyroid RFA?
3. What were the results of implementing thyroid RFA?
   1. Probe: For yourself?
   2. Probe: For your patients?
   3. Probe: For the healthcare system?
4. What advice or recommendations would you provide to those interested in pursuing thyroid RFA?

Barriers and Facilitators:

1. What do you perceive as the main barriers to adopting thyroid RFA in Canada?
   1. Probe: Are there challenges related to training availability?
   2. Probe: Do you believe the cost of equipment and overhead costs is a barrier?
   3. Probe: How does lack of public coverage for thyroid RFA impact adoption?
   4. Probe: Is there patient or institutional resistance to the procedure?
2. What factors would facilitate the adoption of thyroid RFA in Canada?
   1. Probe: How important is collaboration with early adopters or experienced practitioners (peer support)?
   2. Probe: How does leadership support or lack thereof impact the adoption of thyroid RFA?
3. Do you think RFA could help ease the strain on health systems, such as operating-room time or reducing patient wait times?
4. What is your perception of the availability of training opportunities for thyroid RFA?
   1. Probe: How accessible are these resources for physicians in Canada?

Public Coverage as a Key Barrier:

1. How important do you think public coverage for thyroid RFA is for increasing treatment access for patients?
   1. Probe: Where do you think your province is at with public coverage for RFA?
2. What type of coverage do you think RFA should fall under?
   1. Probe: Fully out of pocket, hybrid, or completely covered?

Feasibility:

1. How feasible was it to implement thyroid RFA in your practice or organization?
   1. Probe: Were there institutional policies or logistical issues that might affect adoption?
   2. Probe: How do equipment costs and lack of public coverage impact feasibility?
2. What changes in funding or healthcare policy could make thyroid RFA adoption more viable?
3. Do you see RFA becoming a standard option for certain thyroid nodules in Canada?
   1. Probe: Are there specific patient populations who would benefit most from RFA?
   2. Probe: Do you have any reservations about thyroid RFA?

Health Equity:

1. How do you think the adoption of thyroid RFA could impact health equity in thyroid care across Canada?
2. What policy changes or funding initiatives could ensure equitable access to thyroid RFA?

Conclusion:

1. Do you have any other concluding thoughts about thyroid RFA?
